# Supplementary material for: ONX 0914 Lacks Selectivity for the Cardiac Immunoproteasome in CoxsackievirusB3 Myocarditis of NMRI Mice and Promotes Virus-Mediated Tissue Damage
Source: Cells. 2020 Apr 28;9(5):1093. doi: 10.3390/cells9051093 (PMC7290815; doi:10.3390/cells9051093)
Supplement: Supplementary file 1 [file cells-09-01093-s001.pdf]

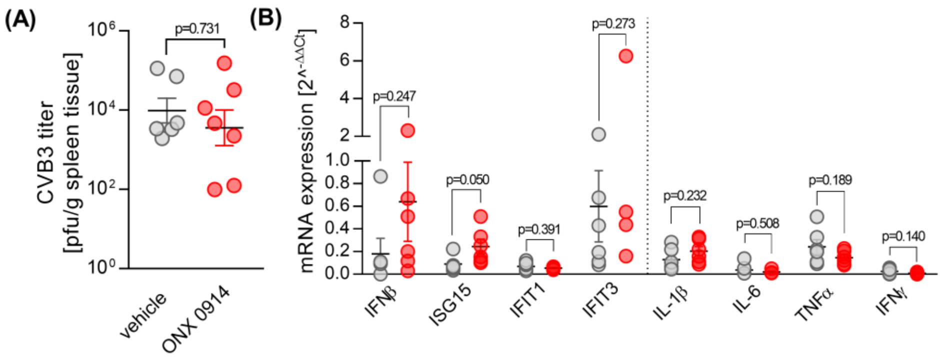

Figure S1. Influence of ONX 0914 on the inflammatory response in spleen after CVB3 infection. Mice received either ONX 0914 or vehicle (10mg/kg BW) daily until day 8, starting one day prior to infection with 5 x 10^5 pfu of CVB3 strain 31-1-93. We determined CVB3 viral titers in spleen tissue from mice treated with ONX 0914 or the vehicle prophylactically and presenting acute cardiac inflammation. Splenic tissue was homogenized, diluted step-wise and used for standard plaque assay to determine viral pfu/g tissue (**A**). Interestingly, and contrasting the effects shown on heart muscle tissue in *Figure 3*, viral titers in spleen tissue did not differ between treatment groups. (**B**) Looking at the mRNA expression of pro-inflammatory cytokines and the IFN response in spleen, we found no significant differences between treatment groups. Data are mean ± SEM and were analyzed using unpaired t-tests. P-values are indicated.

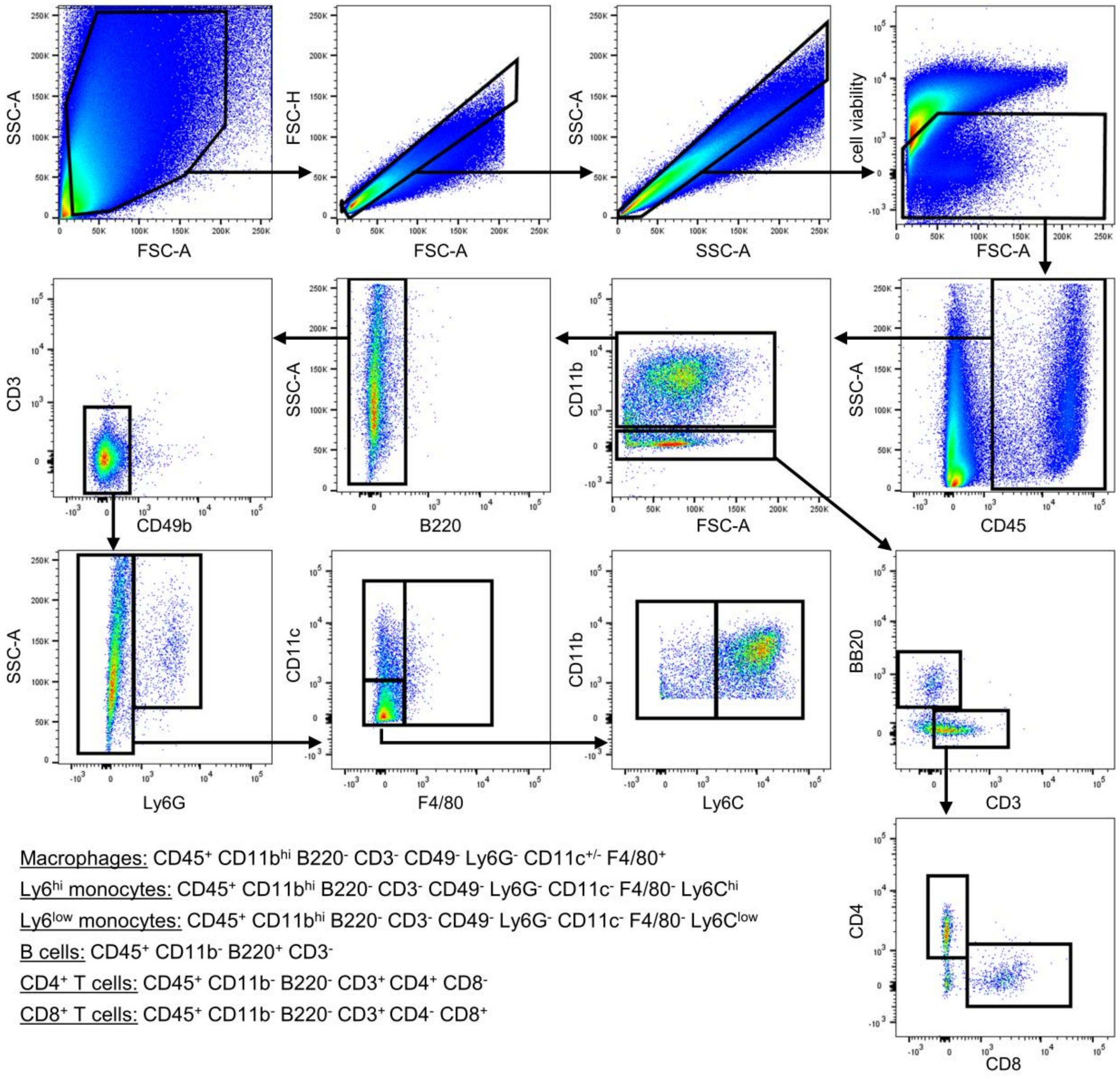

Figure S1. Gating strategy for flow cytometric analysis of heart tissue. Representative flow cytometry plots of cardiac cell suspensions showing the sequential gating scheme for immune cell phenotyping. Following side scatter (SSC) and forward scatter (FSC) discrimination, doublet cells and dead cells were excluded from the CD45^{+} cell subset. Subsequently, macrophages, Ly6C^{hi} and Ly6C^{low} monocytes, B cells, CD4^{+} and CD8^{+} T cells were defined by the expression of the indicated immunological markers.
